# Comparing Patient Comfort During Bronchoscopy Under Conscious Sedation and Monitored Anesthesia Care: A Prospective, Observational, Controlled Study

**DOI:** 10.7759/cureus.62381

**Published:** 2024-06-14

**Authors:** Syed Murtaza Hassan Kazmi, Mahnoor Nawaz Abbasi, Yusra Mudassir, Rashiqua Sulman Chaudhary, Ayesha Siddiqa, Muslim Atiq, Syed Shah Hussain Jafry, Anum Ilyas

**Affiliations:** 1 Pulmonology, Shifa International Hospital Islamabad, Islamabad, PAK; 2 General Medicine, Shifa International Hospital Islamabad, Islamabad, PAK; 3 Respiratory Therapy, Shifa International Hospital Islamabad, Islamabad, PAK; 4 Repiratory Therapy, Shifa International Hospital Islamabad, Islamabad, PAK; 5 Gastroenterology and Hepatology, Shifa International Hospital Islamabad, Islamabad, PAK; 6 Medicine, Jinnah Sindh Medical University, Karachi, PAK; 7 Critical Care Unit, Shifa International Hospital Islamabad, Islamabad, PAK

**Keywords:** sedation depth, comfort levels, monitored anesthesia care (mac), conscious sedation, bronchoscopy

## Abstract

Background: Flexible bronchoscopy (FB) often involves sedation, with the choice left to the bronchoscopist's discretion. Prior research on sedation in gastroscopic endoscopies yields conflicting information regarding the preferred method for FB. This study compares patient comfort levels during bronchoscopy with mindful sedation using fentanyl, nalbuphine, and midazolam versus monitored anesthesia care (MAC) using propofol, midazolam, and ketamine.

Methods: This prospective observational study assessed 83 patients undergoing bronchoscopy under either conscious sedation (CS) (n=40) or MAC (n=43). Patient comfort, sedation levels, emotional state, recovery time, safety, and the impact of smoking history and comorbidities were evaluated. Data collection included direct patient questioning and observation using the Modified Observed Assessment of Alertness and Sedation (MOAA/S) form.

Results: Comfort levels were similar between groups, with mean scores of 3.6±0.89 for CS and 3.3±0.54 for MAC. MAC induced deeper sedation (mean scores: 4.37±0.66 vs. 3.8±0.98). Recovery time and complications were comparable. Emotional states and medical history did not significantly differ between groups.

Conclusion: CS is not inferior to MAC for bronchoscopy, providing comparable comfort and safety with less intense sedation and lower cost. These findings support the use of CS for bronchoscopy procedures, offering a cost-effective alternative without compromising patient comfort or safety.

## Introduction

The primary goal of healthcare is patient comfort, and it is the duty of physicians to select methods that minimize procedural discomfort and ensure positive outcomes. Sedation techniques, such as reducing anxiety and pain and providing amnesia, can make diagnostic and therapeutic procedures more tolerable for patients [1].

With a range of suitable sedation choices, bronchoscopy is a frequently used analytical and therapeutic tool in medicine. Flexible bronchoscopy (FB) is often conducted under a sedative with the sedative chosen at the discretion of the bronchoscopist [2].

Although previous studies have assessed and compared different sedation levels in gastroscopic endoscopies, they have not adequately addressed comfort as a primary outcome in bronchoscopy studies [3]. Additionally, there are conflicting data regarding the preferred sedation type during flexible bronchoscopy. Although deep sedation is generally recommended for patients undergoing endobronchial ultrasound with transbronchial needle aspiration (EBUS-TBNA), moderate sedation may also be acceptable [3-5].

A South Korean meta-analysis found that moderate sedation during FB was associated with an increased willingness to repeat the procedure and a shorter procedural duration [6]. Furthermore, there were no more complications with moderate sedation than with no sedation, such as hypoxic events and significant desaturation.

A recent Italian study suggested that conscious sedation (CS) is a viable and well-tolerated option for EBUS-TBNA procedures [7]. The diagnostic rate of this method, which uses midazolam and meperidine, is comparable to that documented in the literature. A study conducted in New Delhi also assessed the risk-benefit score for children undergoing FB, indicating that propofol provides a shorter sedation induction time, reduced coughing during the procedure, and shorter recovery time than fentanyl [8]. Chrissian and Bedi reported that procedurally delivered medium sedation is safer and more cost-efficient while maintaining diagnostic yield and efficacy in EBUS-TBNA [9]. Previous studies have also sought to evaluate complications related to bronchoscopy to improve patient comfort [10]. Skinner's study examined how comfortable the patients were during bronchoscopy in two settings: conscious sedation and general anesthesia [11]. The results demonstrated that CS, albeit milder, did not result in reduced levels of comfort. Patients who underwent CS also had fewer difficulties. Furthermore, the accuracy of diagnosis of both techniques was comparable. While previous literature suggests that moderate sedation generally offers better outcomes than deeper sedation, further analysis is needed to determine whether CS improves patient comfort compared with moderate anesthesia coverage during bronchoscopy [12-14].

The study aimed to compare ease for patients during bronchoscopy with CS with fentanyl, nalbuphine, and midazolam against moderate anesthesia with propofol, midazolam, and ketamine.

## Materials and methods

This prospective, observational study was conducted at Shifa International Hospital's bronchoscopy suite over a period from October 2022 to August 2023. The study protocol received approval from the institutional review board (IRB #0301-22), and all participants provided informed consent.

The primary objective of the study was to evaluate the difference in patient comfort levels during bronchoscopy under two sedation methods: CS and monitored anesthesia care (MAC). Secondary objectives included assessing differences in sedation levels, emotional state, recovery time, complications, and the impact of smoking history and comorbidities on the procedure.

Eligible patients were those aged 18 to 79 years, classified as risk classes I to III by the American Society of Anesthesiologists (ASA). Exclusion criteria included mental illnesses, females of childbearing age, allergies to soy or anesthetic drugs, severe chronic obstructive pulmonary disease requiring oxygen therapy, unstable hemodynamic status (characterized by heart rate below 60 or above 120 beats per minute and/or systolic blood pressure below 100 or above 180 mmHg), and problematic upper airways indicated by a Mallampati classification score of III or IV.

Patients were divided into two groups based on the anesthesia method selected by the performing physician. All participating physicians had over 20 years of experience, ensuring a high level of expertise in their decision-making. This method of grouping aimed to minimize potential selection bias and maintain consistency in the selection criteria for anesthesia.

CS was administered by a proceduralist assisted by a staff nurse and an associate technician, without the presence of an anesthetist. The sedation protocol involved fentanyl and midazolam, titrated to a maximum of 0.1 mg and 5 mg, respectively. In contrast, MAC was performed by an anesthetist using anesthetic agents such as propofol, ketamine, xylocaine, nalbuphine, and midazolam, chosen at the anesthetist's discretion.

Data collection was both subjective and objective. Subjective data were obtained through direct patient questionnaires administered before and after the procedures. The level of patient comfort was evaluated using a 10-point Likert Scale as adapted from Skinner et al., where 1 represented complete tolerability and 10 represented complete intolerability [11]. Patients responded to the questionnaire within 48 hours post-procedure.

Objective data were collected by staff members who documented sedation levels using the Modified Observed Assessment of Alertness and Sedation (MOAA/S) form during the procedure. Recovery time and complications were recorded as secondary outcomes. Emotional states were assessed pre- and post-procedure using an adapted questionnaire from Skinner et al. that measured periprocedural anxiety and emotional state scores by subtracting the negative emotion states (stressed, unhappy, and anxious) from the positive emotion states (relaxed, comfortable, and satisfied) [11].

The study utilized a power analysis to establish the minimal sample size needed for a two-tailed paired samples t-test, aiming for a power statistic of at least 0.95 and an alpha of 0.05, using a medium effect size (d=0.50) based on data from a previous study [11]. Ultimately, the study examined 83 participants. Quantitative parameters are reported as mean ± standard deviation (SD), with variations among groups treated and 95% confidence levels. Qualitative variables were contrasted using the t-test, chi-square test, or Fisher's exact test, as applicable, with all tests being two-sided. Statistical significance was determined at p < 0.05. Subject comfort levels were assessed using matched-pair analysis and Pearson's correlation coefficient.

The investigation adhered to the ethical guidelines of Shifa International Hospital, Shifa Tameer-e-Millat University, and the Shifa Clinical Research Center.

## Results

The present study comprised 145 patients; however, after applying the exclusion criteria, a sample size of 83 subjects was obtained. Among these individuals, 40 (48.2%) received CS, and 43 (51.8%) received MAC (Table 1).

**Table 1:** Distribution of patients according to the procedure type (n=83) CS: Conscious sedation; MAC: monitored anesthesia care

Procedure Type	Frequency	Percentage
CS	40	48.2
MAC	43	51.8

There were no statistically notable variations in age, gender, Charlson comorbidity index, and smoking history as illustrated in Table 2. All CS procedures were carried out with fentanyl, nalbuphine, and midazolam. The most common supervised anesthesia treatment strategies included opioids 28 (65.0%), propofol 34 (80.0%), and midazolam 26 (60.7%).

**Table 2:** Characteristics of participants CS: Conscious sedation; MAC: monitored anesthesia care

Characteristics	CS (n=40)	MAC (n=43)	p-value
Age	45.83 ± 17.81	47.56 ± 18.96	0.674
Gender	18 (45%) males	21 (48.84%) males	0.897
Charlson Comorbidity Index	3.67 ± 1.41	4.2 ± 1.51	0.09
Smoking History	18 (45%)	21 (48.84%)	0.897

An assessment of the level of comfort experienced by subjects undergoing both types of procedures was conducted by administering a tolerability questionnaire in the recovery room after the procedure. Participants were asked to rate their comfort level on a 10-point Likert Scale, with 1 representing complete tolerability and 10 representing complete intolerability. The mean comfort score reported by subjects was 3.6±0.89 and 3.3±0.54 (95% CI- 3.3- 3.9) on a 10-point scale in the CS and MAC groups, respectively. Based on the non-inferiority criterion of ± 1.5, there was no major statistical variation in subject comfort during the procedure between the two groups, as demonstrated by the p-value of 0.218. Therefore, it can be concluded that there was no difference in subject comfort during the procedure as determined by the tolerability criteria (Table 3).

**Table 3:** Level of comfort on a 10-point Likert Scale according to the procedure type MAC: monitored anesthesia care

Procedure Type	Mean	N	Std. Deviation	p-value
Conscious Sedation	3.65	40	0.89299	0.218
MAC	3.3953	43	0.5407	
Total	3.5181	83	0.73851	

In this study, 83 patients underwent objective evaluation of their sedation levels before and after the two procedures. The sedation level was determined using the MOAA/S tool, which was completed by nursing staff, bronchoscopists, anesthetists, or assistant technicians. The patient’s sedation level was reported using a scale of 1 to 6, with 6 indicating that the patient was alert and awake and readily responding to a name spoken in a normal tone and 1 indicating that the patient did not respond to noxious stimuli.

The results of the MOAA/S assessment were cross-tabulated with the procedure type and revealed that the mean sedation level was 4.37±0.66 in the CS group and 3.8±0.98 in the MAC group. This difference was statistically significant (p=0.008), indicating that patients in the MAC group received a deeper level of sedation compared to those in the CS group.

**Figure 1:**
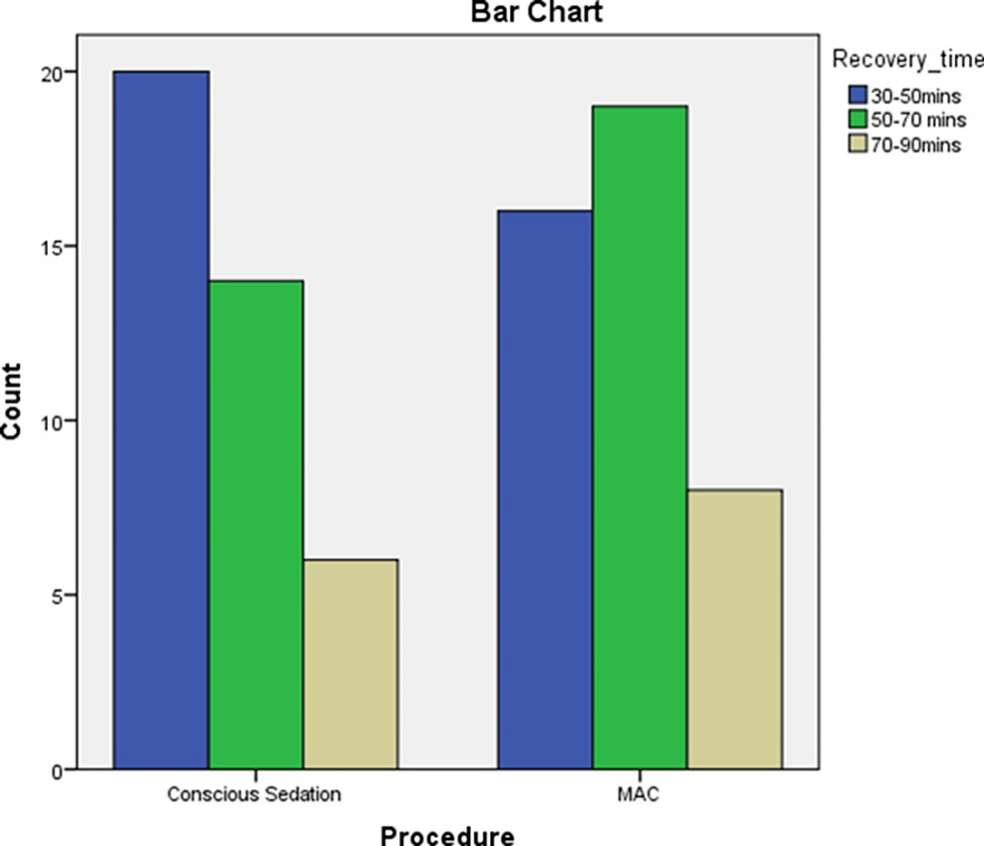
Range of recovery time across the procedure type MAC: Monitored anaesthesia Care p=0.631

The comparison of recovery times between the CS and MAC groups shows no statistically significant difference (p=0.631) (Figure 1).

The study revealed that there was no significant difference in complications between CS and MAC for bronchoscopy procedures. The mean complication rate was 1.9±0.3 in the CS group and 1.9±0.29 in the MAC group (p= 0.914). The safety of the procedure can be adequately assessed by examining the resulting complications, as both procedures demonstrated minimal complications. However, hypotension, bradycardia, and arrhythmias were more frequently associated with MAC, whereas excessive cough, desaturation, hypertension, and tachycardia were more commonly observed during CS. These findings indicate that bronchoscopy is equally safe under CS and MAC, with regard to complications. 

Furthermore, the study assessed individuals' emotional states before and after the procedure using questionnaires administered in the bronchoscopy suite. These questionnaires were either filled directly by the subjects or by personnel for patients who were unable to write. The survey measured periprocedural anxiety, and the emotional state score was computed by deducting negative emotion ratings (stressed, unhappy, and anxious) from positive emotion scores (relaxed, comfortable, and satisfied). The findings demonstrated non-significant variation in the patients' periprocedural emotional states across both types of procedures (Table 4).

**Table 4:** Mean emotional state scores between the two groups MAC: Monitored anesthesia care

Emotional State Score	Conscious Sedation	MAC	p-value
Pre-procedure	1.0±0.1	0.5±0.1	0.09
Post-procedure	0.4±0.01	0.3±0.01	0.09

Moreover, the study accounted for comorbidities and smoking status while stratifying the patients into two groups. The history of diabetes, hypertension, and smoking status was equally prevalent in both groups and did not affect procedure comfort. Furthermore, age and sex did not influence comfort outcomes under either procedure.

## Discussion

In this study, we assessed the efficacy of MAC versus CS in bronchoscopy, with a focus on patient comfort, sedation depth, and recovery. Our findings indicate no significant difference in patient comfort between MAC and CS, aligning with prior research indicating the complexity of sedation choices in bronchoscopic procedures and their impact on patient outcomes [15]. Both sedation methods offered satisfactory comfort, with MAC providing a deeper sedation level, a finding that is consistent with the inherent design of MAC to provide a deeper and more controlled sedation [16]. 

The effectiveness of MAC, especially in procedures outside the operating room, has been highlighted for its safety, comfort, and rapid recovery, qualities that are essential for the diverse needs of bronchoscopy patients [16]. This corroborates our observation that MAC can be an effective option for bronchoscopy, offering a balance between adequate sedation and patient safety. Furthermore, the study by Zhou et al. on using target-controlled infusion remifentanil with dexmedetomidine for MAC during bronchoscopy in patients with severe tracheal stenosis supports the tailored approach of MAC in providing effective sedation while maintaining spontaneous breathing, an aspect crucial for the safety of patients with complex respiratory conditions [17].

Our results also highlight the importance of selecting the right sedation technique based on individual patient needs and procedural requirements, a decision-making process that benefits from a thorough understanding of the advantages and limitations of each sedation method. For instance, the study by Talih et al. comparing MAC and general anesthesia in endobronchial coil treatment highlighted the benefits of MAC, such as reduced remifentanil consumption and shorter recovery duration, underscoring MAC's potential advantages in specific procedural contexts [18].

In conclusion, this study and current literature provide evidence supporting the use of both CS and MAC in bronchoscopy, emphasizing the need for individualized patient care. While MAC offers deeper sedation, it does not significantly improve patient comfort compared to CS [19]. Both methods are safe and effective, with the choice between them depending on specific patient and procedural factors. Future studies should continue to explore the nuanced benefits and limitations of each sedation method to further refine bronchoscopy practice.

However, this study has a few limitations. Evaluating patient comfort after anesthesia is inherently difficult because of the amnesiac impact of sedatives, making subjective patient assessments the most commonly used method in previous research. The study complied with suggestions to do assessments 48 hours after the procedure to improve the dependability of the patient replies. Furthermore, bias could not be totally eliminated because the bronchoscopist was free to choose the type of anesthesia. As a result, the proceduralist or anesthetist had some knowledge of the participants' sedation choice.

## Conclusions

In light of the findings of this study, it can be safely concluded that CS is not inferior to MAC in all patients undergoing bronchoscopy. The results indicate that CS provides an equal level of comfort as MAC and that it is equally safe with no substantial variation in the frequency of complications or duration of recovery among the two groups. Additionally, CS provides less depth of sedation and is more cost-effective, as it requires less manpower compared to MAC. Consequently, this study supports and encourages the use of CS during bronchoscopies.
